# Volatile compounds as potential bio-fumigants against plant-parasitic nematodes – a mini review

**DOI:** 10.21307/jofnem-2021-014

**Published:** 2021-02-15

**Authors:** Hung Xuan Bui, Johan A. Desaeger

**Affiliations:** Department of Entomology and Nematology, Gulf Coast Research and Education Center, University of Florida, Wimauma, FL, 33598

**Keywords:** Bio-fumigant, Volatile compounds, Plant-parasitic nematodes, Biological control, Nematode management

## Abstract

Soil fumigation remains the standard practice to manage soilborne pathogens such as plant-parasitic nematodes, bacteria, and fungi, especially in high-value crops. However, increasing regulatory pressure due to the inherent and broad-spectrum toxicity and negative environmental impact of chemical soil fumigants, its negative effect on overall soil health, and increasing demand for organic produce, has created a growing interest in biological fumigants. Many plants and microorganisms emit volatile compounds, which can potentially be used as bio-fumigants. In this mini-review, we summarize the current status of nematology studies focused on the development of volatile compounds emitted from plants and microorganisms as fumigants to control plant-parasitic nematodes. The gap of knowledge and challenges of studying volatile compounds are also addressed.

Plant-parasitic nematodes (PPNs) are one of the major constraints to crop production, and especially in high-value vegetable and fruit crops, they can cause significant economic yield loss, estimated to be more than US$100 billion annually (Bernard et al., 2017). Chemical soil fumigants have been in use for more than a century now, and remain the standard practice in many crops, especially fruits and vegetables. Although many of the early fumigants have been banned, the ones that have managed to stay, such as 1,3-dichloropropene, metam, and chloropicrin, are still considered to be the most effective products for the control of PPNs (De Cal et al., 2005; Desaeger et al., 2017; Rosskopf et al., 2005). However, environmental and safety concerns are putting more and more pressure on these products. Also, the evidence is growing of their adverse effect on beneficial soil organisms and the rapid resurgence of soilborne pathogens, including PPNs, following fumigation (Dangi et al., 2017; Martin 2003; Mazzola et al., 2015; Raupach and Kloepper, 2000; Sánchez-Moreno et al., 2010; Watson et al., 2017). As limitations of chemical soil fumigants are becoming more apparent, there is a need to find new soil fumigation compounds that are safer for the soil ecosystem and the environment. In recent years, volatile compounds (VCs) emitted from plants and microorganisms have been increasingly studied as bio-fumigant candidates for the control of various soilborne pathogens, including PPNs. Effects of VCs on plants, and soilborne pathogens such as bacteria and fungi have been reviewed elsewhere (Kai et al., 2009, 2016; Schulz-Bohm et al., 2017). Here, we summarize the recent studies of VCs that focused on PPNs as well as the challenges and knowledge gaps that remain in the future application of VCs as potential bio-fumigants for nematode management in the field.

## What are volatile compounds?

Volatile compounds (VCs) are typically small, lipophilic, odorous, and low molecular mass compounds that can be evaporated and diffused aboveground and belowground through gas- and water-filled pores in soil and rhizosphere environments (Effmert et al., 2012; Insam and Seewald, 2010; Vespermann et al., 2007). These VCs are considered as the products of secondary metabolisms in plants and microorganisms such as bacteria and fungi (Dudareva et al., 2013; Schulz-Bohm et al., 2017; Vivaldo et al., 2017). The emission of VCs from plants and microorganisms depends on various factors such as the growth stage, nutrient availability, temperature, oxygen availability, pH, and soil moisture content (Insam and Seewald, 2010). VCs are classified into different chemical classes such as alkenes, alcohols, ketones, benzenoids, pyrazines, sulfides, and terpenes which have either beneficial or harmful effects on other organisms (Schmidt et al., 2015; Vivaldo et al., 2017).

## Plant volatile compounds against plant-parasitic nematodes

Plant VCs that are well-known as bio-fumigants for the control of PPNs are glucosinolates which emit isothiocyanates (ITCs) as VCs under the process of biodegradation. ITCs are also the active ingredient of chemical fumigants such as metam. Plants belonging to the families Brassicaceae, Capparaceae, and Caricaceae all produce glucosinolates, and many genera within these plant families have been studied for their nematicidal effects on PPNs (Kruger et al., 2013; Monfort et al., 2007) (Table 1). Following maceration and incorporation, glucosinolates will be hydrolyzed to release ITCs which have broad-spectrum biological activities, against many soilborne pathogens and PPNs (Matthiessen et al., 2004; Schroeder and MacGuidwin, 2010). Several studies have shown the potential of these plants to control PPNs such as *Meloidogyne incognita*, *M. javanica*, *Heterodera schachtii*, *Pratylenchus neglectus*, *Paratrichodorus allius*, and *Globoderra pallida* (Lord et al., 2011; Potter et al., 1998; Thierfelder and Friedt, 1995). More than 200 glucosinolates were identified from 3,500 Brassica species and each Brassica species can contain various types and amounts of glucosinolates (Clarke, 2010). Additionally, more than 120 glucosinolates were identified from at least 500 non-Brassica plants (Kruger et al., 2013). The use of Brassica and non-Brassica plants as biofumigation crops can be through maceration and incorporation of plant parts into the soil as green manure, through the use of seed meal, or as poor-host winter or summer cover crops (Hafez and Sundararaj, 2009; Rahman and Somers, 2005; Smith et al., 2004). Biofumigation is usually not as effective as chemical fumigation and biofumigant crops can also be good hosts to some of the target PPNs (Daryanto et al., 2018; Grabau et al., 2017; Monfort et al., 2007). The cost of biofumigation is still high and often not economically practical for farmers to apply (Clay et al., 2020; Dutta et al., 2019). There are excellent review articles of using Brassica plants as biofumigation to control PPNs that readers can find in the literature (Brennan et al., 2020; Dutta et al., 2019).

**Table 1.** Overview of studies investigating the effect of plant volatile compounds on different plant-parasitic nematodes.

Volatile compound producers	Identified volatile compounds	Experiment conditions	Target plant-parasitic nematodes	References
Brassica	Glucosinolates and isothiocyanates	Field biofumigation	*Meloidogyne incognita*, *M. javanica*, *Heterodera schachtii*, *Pratylenchus neglectus*, *Paratrichodorus allius*	Thierfelder and Friedt (1995), Potter et al. (1998), Lord et al. (2011)
White mustard (*Sinapis alba*)	Methyl sulfide, dimethyl disulfide	Field biofumigation	*Tylenchulus semipenetrans*	Wang et al. (2009)
Brassica leaf	2-propenyl glucosinolate	Field biofumigation	*Globodera pallida*	Lord et al. (2011)
*Brassica juncea*, *Azadirachta indica*, *Canavalia ensiformis*, *Mucuna pruriens*, and *Cajanus cajan*	Alcohols and esters and sulfur containing compounds (mainly isothiocyanates)	In vitro and greenhouse	*M. incognita*	Barros et al. (2014)
Camellia seed cake	18 compounds were identified	In vitro	*M. javanica*	Yang et al. (2015)
*Eruca sativa*	(Z)-3-hexenyl acetate, (Z)-3-hexen-1-ol and erucin	In vitro	*M. incognita*	Aissani et al. (2015)
Cottonseed meal	2-methyl-1-butanol, 3-methyl-1-butanol, phenyl-ethylalcohol, benzene-1-ethyl-4methoxy (p-ethylanisole), and 4-ethyl-1,2-dimethoxybenzene	In vitro and greenhouse	*M. incognita*	Estupiñan-López et al. (2017)
Castor bean cake	Phenol, 4-methylphenol, γ-decalactone, and skatole	In vitro and greenhouse	*M. incognita*	Pedroso et al. (2019)
Citronella (*Cymbopogon nardus* L.) or black pepper (*Piper nigrum* L.) leaves, broccoli shoots (*Brassica oleracea* L.) and Brazil nuts (*Bertholletia excelsa* Bonpl.)	Dimethyl disulfide and 3-pentanol	In vitro and greenhouse	*M. incognita*	Silva et al. (2018), da Silva et al. (2019)
Watercress (*Nasturtium officinale*) leaves and passion fruit (*Passiflora edulis*) seeds	26 and 12 compounds were identified. 1-octanol had strong nematicidal activity	In vitro and greenhouse	*M. incognita*	Silva et al. (2020)
Seeds of papaya fruit (*Carica papaya*)	Vinyl acetate, phenylacetaldehyde and benzylacetonitrile	In vitro and greenhouse	*M. incognita*	Gomes et al. (2020)
*Cymbopogon nardus* and *Dysphania ambrosioides*	Ascaridole and citronellal	In vitro and greenhouse	*M. incognita*	de Freitas Silva et al. (2020)

Recently, many other plant VCs have been shown to have potential for controlling PPNs. Dimethyl disulfide and 3-pentanol, selected from the broccoli volatilome, were able to reduce the mobility of *M. incognita* in vitro and gall incidence and egg production on tomato *in planta* (da Silva et al., 2019; Silva et al., 2018). Interestingly, the dry macerates of citronella, black pepper, and broccoli worked more effectively than the aqueous filtered macerates (da Silva et al., 2019). Phenol, 4-methylphenol, γ-decalactone, and skatole emitted by castor bean cake inhibited egg hatching and mobility and caused mortality to second-stage juveniles (J2s) of *M. incognita*. Also, *M. incognita* J2s exposed to these VCs showed reduced infectivity and reproduction on tomato (Pedroso et al., 2019). 2-methyl-1-butanol, 3-methyl-1-butanol, phenyl-ethylalcohol, benzene-1-ethyl-4-methoxy (p-ethylanisole), and 4-ethyl-1,2 dimethoxybenzene are the main VCs emitted from cottonseed meal and immobilized 95 to 100% of *M. incognita* J2s after 20 days of exposure and reduced gall formation and eggs on tomato in a greenhouse trial (Estupiñan-López et al., 2017). (Z)-3-hexenyl acetate, (Z)-3-hexen-1-ol and erucin selected from rucola (*Eruca sativa*) volatilome killed *M. incognita* J2s in an in vitro test (Aissani et al., 2015).

The VCs ascaridole and citronella, emitted from two medicinal plants citronella grass (*Cymbopogon nardus*) and Mexican tea (*Dysphania ambrosioides*), immobilized 46 to 79% of *M. incognita* J2s in vitro and reduced 19 to 37% of gall formation and 80% of eggs on tomato under greenhouse conditions (de Freitas Silva et al., 2020; Silva et al., 2020). Seeds of papaya fruit (*Carica papaya*) emitted VCs that killed 80% of *M. incognita* J2s in vitro and reduced root galls and nematode eggs by 70%. VCs from papaya seed were identified as vinyl acetate and phenylacetaldehyde (Gomes et al., 2020). Certainly, these results indicate that many plants can produce VCs that have nematicidal activity, and probably many more remain to be identified.

## Fungal volatile compounds against plant-parasitic nematodes

Several fungal VCs have been evaluated against PPNs, mostly *Meloidogyne* spp. (Table 2). Non-pathogenic *Fusarium oxysporum* not causing disease to plants have been used as biocontrol agents due to their suppression of plant pathogens such as Fusarium wilt (*Fusarium oxysporum*) and Verticillium wilt (*Verticillium dahliae*) on a wide host range including various vegetables, fruit, and ornamental trees (Mulero-Aparicio et al., 2019; Sajeena et al., 2020). Until now, VCs emitted from *Fusarium* spp. have been the most extensively studied. Among 35 fungi isolated from the coffee plant rhizosphere, including from *Meloidogyne exigua* eggs and egg masses on coffee roots, *Fusarium oxysporum* isolates 20a and 21 and an *F. solani* isolate caused 88 to 96% *M. incognita* J2 mortality. Also, *M. incognita* J2s lost their infectivity when exposed to *F. oxysporum* isolate 21 VCs which were identified as dioctyl disulfide; caryophyllene; 4-methyl-2,6-di-tert-butylphenol; and acoradiene (Freire et al., 2012). Another *F. oxysporum* isolate 26 isolated from *M. exigua* egg masses caused 94% immobility and 27% mortality to *M. exigua* J2s in vitro. However, the VCs were not identified in this study (Costa et al., 2015). In the studies of Terra et al. (2017, 2018), *Fusarium oxysporum* strain 21 was used to test the VCs effectiveness against *M. incognita*. The results indicated that VCs from *Fusarium oxysporum* strain 21 immobilized 100% of *M. incognita* J2s and reduced the infectivity of *M. incognita* J2s and reproduction by 70 and 65%, respectively. More than 28 VCs were identified in which 2-methylbutyl acetate, 3-methylbutyl acetate, ethyl acetate, and 2-methylpropyl acetate killed 80 to 100% of *M. incognita* J2s. Also, 3-methylbutyl acetate and ethyl acetate inhibited 90% of *M. incognita* egg hatching (Terra et al., 2017). However, only 2-methylbutyl acetate reduced gall formation by 22% compared to the control. Estupiñan-López et al. (2018) showed that VCs emitted from *F. oxysporum* isolate F63 and *F. solani* isolate F12 isolated from *M. paranaensis* egg masses caused 100% and 40 to 70% of immobility to *M. incognita* J2s in vitro at 25°C in the dark for six days, respectively. More than 50% of gall and egg reduction has been observed when *M. incognita* J2s were exposed to water containing fungal VCs prior to inoculation to tomato plants.

**Table 2.** Overview of studies investigating the effect of fungal volatile compounds on different plant-parasitic nematodes.

Volatile compound producers	Identified volatile compounds	Experiment conditions	Target plant-parasitic nematodes	References
*Muscodor albus*	Unidentified	In vitro	*Meloidogyne chitwoodi, M. hapla, Paratrichodorus allius*, and *P. penetrans*	Riga et al. (2008)
*Trichoderma* sp. YMF 1.00416	1β-vinylcyclopentane-1α,3α-diol, 6-pentyl-2H-pyran-2-one and 4-(2-hydroxyethyl) phenol	In vitro	*Bursaphelenchus xylophilus*	Yang et al. (2012)
*Fusarium oxysporum* and *Fusarium solani*	Dioctyl disulfide (2-propyldecan-1-ol or 1-(2-hydroxyethoxy) tridecane); caryophyllene; 4-methyl-2,6-di-tert-butylphenol; and acoradiene	In vitro and greenhouse	*M. incognita*	Freire et al. (2012)
*Fusarium oxysporum*	Unidentified	In vitro	*M. exigua*	Costa et al. (2015)
Endophytic fungus *Daldinia cf. concentrica*	3-methyl-1-butanol, (±)-2-methyl-1-butanol, 4-heptanone, and isoamyl acetate,	In vitro and greenhouse	*M. javanica*	Liarzi et al. (2016)
*Epicoccum nigrum* and *Schizophyllum commune*	Alcohols, esters, terpenes, and ketones	In vitro and greenhouse	*M. incognita*	Pimenta et al. (2017)
*Fusarium oxysporum* strain 21	More than 28 volatile organic compounds were identified.	In vitro and greenhouse	*M. incognita*	Terra et al. (2017)
*Fusarium oxysporum* strain 21	2-methylbutyl acetate, 3-methylbutyl acetate, ethyl acetate, and 2-methylpropyl acetate	In vitro and greenhouse	*M. incognita*	Terra et al. (2018)
*Fusarium oxysporum* and *Fusarium solani*	23 compounds belong to esters, alcohols, phenols, aldehydes, carboxylic acids and sesquiterpenes	In vitro and greenhouse	*M. incognita*	Estupiñan-López et al. (2018)

*Trichoderma* spp. are well-known as biocontrol agents of many soilborne pathogens and have been studied as biocontrol agents of PPNs as well (Reino et al., 2008; Sharon et al., 2011). However, little is known about VCs from *Trichoderma* spp. VCs from the unidentified *Trichoderma* sp. YMF 1.00416 isolated from soil in Yunnan, China were tested against *Bursaphelenchus xylophilus* in vitro, where 41.53% of *B. xylophilus* were killed (Yang et al., 2012). 1β-vinylcyclopentane-1α,3α-diol, 6-pentyl-2H-pyran-2-one and 4-(2-hydroxyethyl) phenol were identified as the main VCs from *Trichoderma* sp. YMF 1.00416. Among that, 6-pentyl-2H-pyran-2-one was toxic to *B. xylophilus* in 48 hr at 200 mg/L.

The VCs emitted from the endophytic fungus, *Muscodor albus*, isolated from cinnamon tree (*Cinnamomum zeylanicum*) caused more than 80% mortality to J2s of *M. chitwoodi*, *Paratrichodorus allius*, and *Pratylenchus penetrans* after 72 hr of exposure in vitro (Riga et al., 2008). VCs emitted from the endophytic fungus *Daldinia concentrica* (Xylariaceae) have shown the ability to antagonize various fungal pathogens (Liarzi et al., 2016). Volatile compounds from *Daldinia cf. concentrica* isolated from an olive tree (*Olea europaea* L.) in Israel also reduced viability of *M. javanica* J2s by 67%. A mixture of 3-methyl-1-butanol, (±)-2-methyl-1-butanol, 4-heptanone, and isoamyl acetate (1:1:2:1 ratio) based on the VCs emitted from *Daldinia cf. concentrica* demonstrated a 99% reduction of *M. javanica* J2s viability and 87% inhibition of egg hatching. Soil application of this mixture showed a reduction in root galls and egg reproduction on tomato in a greenhouse trial (Liarzi et al., 2016). Another study showed that 28 fungal isolates from decaying wood released toxic VCs that immobilized from 77 to 100% of *M. incognita* J2s (Pimenta et al., 2017). Isolated fungi included *Epicoccum nigrum*, *Schizophyllum commune*, *Pestalotiopsis* sp., *Phanaerochaete chrysosporium*, *Nigrospora* sp., and *Lasiodiplodia sp*. and 20 VCs were identified, including alcohols, esters, terpenes, and ketones.

## Bacterial volatile compounds against plant-parasitic nematodes

A wide diversity of bacterial VCs have been investigated for the suppression of plant pathogens (Audrain et al., 2015; Bennett et al., 2012). However, few studies focused on managing PPNs (Table 3). Gu et al. (2007) investigated VCs of 200 bacterial isolates affecting *B. xylophilus*. Of these, seven isolates had a negative effect on *B. xylophilus* mobility within 24 hr. Among the 20 identified VCs, phenol, 2-octanol, benzaldehyde, benzeneacetaldehyde, decanal, 2-nonanone, 2-undecanone, cyclohexene, and dimethyl disulfide had nematicidal activity on *B. xylophilus*. Similarly, Huang et al. (2010) showed that VCs from *Bacillus megaterium* YFM3.25 inhibited egg hatching and killed *M. incognita* J2s after 24 hr of exposure. Also, these VCs reduced gall formation and egg mass production in a dose-dependent manner. Five bacterial isolates *Pseudochrobactrum saccharolyticum*, *Wautersiella falsenii*, *Proteus hauseri*, *Arthrobacter nicotianae*, and *Achromobacter xylosoxidans* emitted VCs that were toxic to *M. incognita in vitro* within 24 hr. Of the VC identified in these bacterial isolates, S-methyl thiobutyrate, butyl isovalerate, ethyl 3,3-dimethylacrylate, and 1-methoxy-4-methylbenzene showed more than 90% nematicidal activity against *M. incognita* (Xu et al., 2015). VCs from *Pseudomonas putida* BP25 were tested against *Radopholus similis* and showed more than 92% mortality (Agisha et al. 2019; Sheoran et al., 2015). Many VCs were identified such as 1-undecene; disulfide dimethyl; pyrazine, methyl-pyrazine, 2,5-dimethyl-; isoamyl alcohol; pyrazine, and methyl-; dimethyl trisulfide. but the effect of the individual VCs on *Radopholus similis* was not tested. Cheng et al. (2017) also tested the adverse effect of VCs from *Paenibacillus polymyxa* KM 2501 on *M. incognita*. This study showed that the most active VCs to control *M. incognita* J2s were furfural acetone, 2-undecanol, 4-acetylbenzoic, and 2-decanol acid. Zhai et al. (2018) showed that VCs from *Pseudomonas putida* 1A00316 killed almost 100% of *M. incognita* J2s after 72 hr of exposure. From the VC profiles, dimethyl disulfide, 1-undecene, 2-nonanone, 2-octanone, (Z)-hexen-1-ol acetate, 2-undecanone, and 1-(ethenyloxy)-octadecane all inhibited egg hatching of *M. incognita*, and dimethyl disulfide, 2-nonanone, 2-octanone, (Z)-hexen-1-ol acetate, and 2-undecanone also showed nematicidal activity against *M. incognita* J2s. In another report, VCs emitted from *Bacillus* sp., *Paenibacillus* sp. and *Xanthomonas* sp. isolated from soil in a rice field caused more than 99% mortality of *M. graminicola* J2s in vitro and reduced gall incidence and egg production on rice in a greenhouse trial (Bui et al., 2020). However, VC identification from these bacterial isolates was not conducted in this study.

**Table 3.** Overview of studies investigating the effect of bacterial volatile compounds on different plant-parasitic nematodes.

Volatile compound producers	Identified volatile compounds	Experiment conditions	Target plant-parasitic nematodes	References
*Bacillus simplex, B. subtilis, B. weihenstephanensis, Stenotrophomonas maltophilia* and *Serratia marcescens*	Terpineol, benzeneethanol, propanone, phenyl ethanone and nonane	In vitro	*B. xylophilus*	Gu et al. (2007)
*Bacillus megaterium* YFM3.25	Benzeneacetaldehyde, 2-nonanone, decanal, 2-undecanone and dimethyl disulphide,phenyl ethanone, nonane, phenol, 3,5-dimethoxytoluene, 2,3-dimethyl- butanedinitrile and 1-thenyl-4-methoxy- benzene	In vitro and greenhouse	*M. incognita*	Huang et al. (2010)
*Pseudomonas putida, Microbacterium* sp.*, Bacillus methylotrophicus, Bacillus pumilus* and *Bacillus pumilus*	Unidentified	In vitro	*M. exigua*	Costa et al. (2015)
*Pseudomonas putida* BP25	As 1-Undecene; Disulfide dimethyl; Pyrazine, methyl-Pyrazine, 2,5-dimethyl-; Isoamyl alcohol; Pyrazine,methyl-; Dimethyl trisulfide	In vitro	*Radopholus similis*	Sheoran et al. (2015), Agisha et al. (2019)
*Pseudochrobactrum saccharolyticum, Wautersiella falsenii, Proteus hauseri, Arthrobacter nicotianae,* and *Achromobacter xylosoxidans*	Acetophenone, S-methyl thiobutyrate, dimethyl disulfide, ethyl 3,3-dimethylacrylate, nonan-2 one, 1-methoxy-4-methylbenzene, and butyl isovalerate	In vitro	*M. incognita*	Xu et al. (2015)
*Paenibacillus polymyxa* KM 2501	Acetone, 2-heptanone, benzaldehyde, 2-nonanone, 2-nonanol, cyclopentasiloxane, decamethyl-, 11-dodecen-2-one, 2-decanone, 2-decanol, 4-acetylbenzoic acid, furfural acetone, 2-undecanone, Acetic acid, [bis[(trimethylsilyl)oxy]phosphinyl]-, trimethylsilyl ester, 2-undecanol	In vitro	*M. incognita*	Cheng et al. (2017)
*Pseudomonas putida 1A00316*	Dimethyl disulfide, 1-undecene, 2- nonanone, 2-octanone, (Z)-hexen-1-ol acetate, 2-undecanone, and 1-(ethenyloxy)- octadecane. Of these, dimethyl disulfide, 2-nonanone, 2-octanone, (Z)-hexen-1-ol acetate, and 2-undecanone	In vitro	*M. incognita*	Zhai et al. (2018)
*Bacillus* sp., *Paenibacillus* sp. and *Xanthomonas* sp.	Unidentified	In vitro and greenhouse	*M. graminicola*	Bui et al. (2020)

## The gap of knowledge and challenges

There is no standard procedure for testing the effects of VCs on PPNs in vitro. Each study has developed its own device where VCs were kept in closed conditions together with PPNs (two- or three-compartment petri dish, microtube in a vial, or microtube in a closed box). Each design has contributed a valuable test system to the ‘proof of concept’ of the potential use of VCs as bio-fumigants. However, the results might be different when VCs from one source are tested in different designs. Up to now, most of the studies used a two- or three-compartment petri dish for testing the microbial VCs in vitro (Kai et al., 2016). The advantages of this experimental design are simple, inexpensive, and separating the VC emitters and receivers. However, this design also created non-natural conditions that alternated the metabolisms of the tested microorganisms (Kai et al., 2016). For example, the high concentration of CO_2_ accumulation, 10 times higher than the ambient concentration (20°C, 84 μmol m−2s−1 light, 16 h/8 h light/darkness), was the most obvious observation in this design (Kai and Piechulla, 2009). Therefore, standardizing in vitro testing conditions to evaluate the efficacy of VCs is needed. Also, many of the studies did not look at the recovery of nematodes, where following the exposure of VCs, the PPNs are removed from the exposure of VCs and their recovery in the absence of VCs is observed. Also, whether the efficacy is due to an individual VC or a blend of VCs, is often not known.

It is also very important to establish whether VCs are phytotoxic or not. Many researchers have shown that VCs from different microorganisms can actually promote plant growth (Hung et al., 2013; Lee et al., 2014; Nieto-Jacobo et al., 2017; Park et al., 2015; Ryu et al., 2003; Tahir et al., 2017), whereas other studies have shown that VCs from various microorganisms can cause phytotoxicity (Blom et al., 2011; Hung et al., 2013; Lee et al., 2014; Vespermann et al., 2007; Wenke et al., 2012). Recently, Bui et al. (2019) indicated that bacterial VCs inhibited rice germination in vitro but not *in planta*, probably because the concentration of VCs was higher in in vitro conditions than in *in planta* conditions. This obviously applies to nematicidal efficacy as well, as in vitro studies typically employ much higher concentrations than *in planta* studies. Therefore, it is important to keep in mind that in vitro studies, no matter how promising, only indicate potential, and by no means will guarantee that a certain compound will be efficacious in a greenhouse or field. Also, VCs that are phytotoxic are not necessarily bad and may have potential as herbicidal soil fumigants, as long as they are applied sufficiently long before the crop is planted.

Although VCs from plants and microorganisms may constitute a more sustainable approach and reduce the use of synthetic chemical pesticides, potential adverse effects of VCs on human health, the environment and the soil ecosystem also need to be addressed, as biological products are not by definition safer than chemical products. For instance, Bahlai et al. (2010) showed that organic approved insecticides in Canada (Superior 70 oil^®^ (UAP) and Botanigard^®^ (Laverlam)) had more adverse effects on natural enemies (Asian ladybeetle *Harmonia axyridis* and insidious flower bug *Orius insidiosus*) in the laboratory and field conditions than novel synthetic insecticides. However, as plants and microorganisms have been co-existing with humans and emitting VCs for millions of years, it is more likely that VCs emitted from plants and microorganisms are safe for human health, the environment, and the soil ecosystem. For instance, the effects of VCs emitted from the fungus *Muscodor albus* on human health and the environment were studied and no harmful potential was detected (Tilocca et al., 2020).

The mechanisms of VC emission from microorganisms are not clearly understood yet, but some have suggested that VCs are waste products in the microbial lifecycle (Schulz-Bohm et al., 2017). Cheng et al. (2016) and Ossowicki et al. (2017) demonstrated that the production of VCs was triggered by the GaC-A/GaC-S two-component regulatory system in bacteria. New biotechnology techniques such as gene editing may help to better understand the mechanisms of VC emission, and potentially to manipulate microbes to more efficiently release beneficial VCs.

Microbial VCs have also been reported to induce plant resistance to pathogens (He et al., 2006; Huang et al., 2012; Kottb et al., 2015; Lee et al., 2012; Naznin et al., 2014; Park et al., 2013; Raza et al., 2016). In these studies, the mechanism of induced resistance by microbial VCs involves salicylic acid or jasmonic acid/ethylene signaling pathways, similar to the mechanisms of induced resistance by plant growth-promoting microbes in dicot and monocot plants (Balmer et al., 2013; Pieterse et al., 2014). Nonetheless, the exact mechanisms of VCs inducing plant resistance, or their nematicidal mode of action, against PPNs are still unknown. Cheng et al. (2017) suggested that VCs could kill PPNs by affecting the nervous system, surface coat, intestine, pharynx, or other tissues of PPNs. Likely, different VCs have different modes of action as well, and while certain VCs may be nematode-specific, other VCs like isothiocyanates (ITCs), which are produced by glucosinolate-containing plants, are identical to chemical fumigants like metam, and have a broad-spectrum biocidal activity, with a multi-site mode of action.

Currently, several VCs have been shown to be able to control PPNs in the laboratory and sometimes greenhouse conditions. However, field application of VCs is still in its infancy (Farag et al., 2013), and only a few studies have demonstrated success in applying VCs to induce plant resistance against bacterial pathogens and insects on cucumber and pepper under field conditions (Choi et al., 2014; Song and Ryu, 2013). Even if efficacy can be demonstrated in the field, many hurdles remain, not in the least the need to produce or synthesize commercial and cost-effective quantities of VCs. In addition, there will also be a need for technology and equipment to apply VCs, similar to the equipment that is currently used to apply chemical fumigants.

## Conclusions

Evidence is growing that plant and microbial volatile compounds have potential as a more environmentally friendly and ecosystem sustainable alternative to chemical soil fumigants. An increasing number of VCs emitted from plants and microorganisms are studied and have shown nematicidal activity in in vitro and in greenhouse conditions. Field studies are still few and far between, and also the mechanisms of VC emission as well as their effects on host plants, plant-parasitic nematodes, the ecosystem, the environment, and human health are still not well-understood. While we do not claim to have covered all current knowledge, we hope that this review of VCs with regard to PPNs will help to stimulate more research into their use as a potential alternative source of soil fumigants.
